# Adaptation of the RESPECT framework to prevent violence against women, Indonesia

**DOI:** 10.2471/BLT.24.291479

**Published:** 2024-08-18

**Authors:** Alegra Wolter, Yuniyanti Chuzaifah, Aflina Mustafainah, Nunik Nurjanah, Ali A Ramly, Eni Widiyanti, Norcahyo B Waskito, Yuni Asriyanti, Cresti E Fitriana, Sri Wahyuni, Risya Kori, Dirna Mayasari, Agusmia P Haerani, Yasmin Purba, Inga Williams

**Affiliations:** a World Health Organization, Indonesia, 5th floor, Gama Tower, Jl HR Rasuna Said Kav. C-22, 12940 Jakarta, Indonesia.; b United Nations Entity for Gender Equality and the Empowerment of Women, Jakarta, Indonesia.; c United Nations Children’s Fund, Jakarta, Indonesia.; d Ministry of Women Empowerment and Child Protection, Jakarta, Indonesia.; e United Nations Population Fund, Jakarta, Indonesia.; f Joint United Nations Programme on HIV/AIDS, Jakarta, Indonesia.

## Abstract

**Problem:**

Violence against women is a global health problem. Effectively preventing such violence requires locally adapted strategies.

**Approach:**

The World Health Organization in Indonesia, in collaboration with United Nations (UN) Population Fund, UN Women, United Nations Children’s Fund, United Nations Development Programme and Joint United Nations Programme on HIV/AIDS, launched capacity-building initiatives to introduce RESPECT as an evidence-based framework to address violence against women. The initiatives included stakeholder engagement, module development, sensitization training, a workshop and evaluation sessions. These sessions introduced RESPECT strategies to government officials, UN officers and community representatives, mapped best practices and developed action plans.

**Local setting:**

Indonesia has a substantial burden of violence against women, despite numerous laws and policies to combat it. The 2021 Indonesian violence against women survey showed that 26.1% (3341/12 800) of women aged 15–64 years had experienced violence in their lifetime, with 8.7% (1114/12 800) experiencing violence in the past year.

**Relevant changes:**

The initiatives to introduce RESPECT led to securing government support, and verbal commitment was given by the Director for the Protection of Women’s Rights against Domestic Violence and Vulnerability to integrate RESPECT into the 2025–2029 National Development Plan and National Strategy for Reducing Violence Against Women.

**Lessons learnt:**

RESPECT can be adapted to local contexts through customization and capacity-building and by ensuring initial government support, dedicated personnel, resource allocation and use of established relationships with key stakeholders. Improved research, monitoring and evaluation are vital to promote evidence-informed decision-making, together with community engagement and multistakeholder collaboration. The UN can facilitate these efforts.

## Résumé

**Problème:**

La violence à l'égard des femmes est un problème de santé mondial. Lutter efficacement contre de telles violences nécessite des stratégies adaptées aux différents contextes locaux.

**Approche:**

En collaboration avec le Fonds des Nations unies pour la population (FNUAP), l'ONU Femmes, le Fonds des Nations unies pour l'enfance, le Programme des Nations unies pour le développement et le Programme commun des Nations Unies sur le VIH/sida, l'Organisation mondiale de la Santé en Indonésie a lancé une série d'initiatives de renforcement des capacités afin d'instaurer RESPECT, un cadre méthodologique fondé sur des faits dont le but est de lutter contre les violences envers les femmes. Parmi ces initiatives figuraient l'implication des parties prenantes, le développement de modules, une formation à la sensibilisation, un atelier et des sessions d'évaluation. Ces sessions ont servi à présenter les stratégies RESPECT à des responsables du gouvernement, des fonctionnaires de l'ONU et des représentants des communautés, à identifier les meilleures pratiques et à élaborer des plans d'action.

**Environnement local:**

Malgré de nombreuses lois et politiques visant à lutter contre la violence envers les femmes, elle demeure un véritable fléau en Indonésie. Une enquête menée en 2021 dans le pays a montré que 26,1% (3341/12 800) des femmes âgées de 15 à 64 ans avaient subi des violences au cours de leur vie, et que 8,7% (1114/12 800) y avaient été confrontées au cours de l'année écoulée.

**Changements significatifs:**

Les initiatives visant à mettre en place RESPECT ont permis d'assurer le soutien du gouvernement, tandis que le directeur chargé de la protection des droits des femmes contre les violences domestiques et les facteurs de vulnérabilité s'est engagé verbalement à intégrer RESPECT dans le Plan national de développement et la Stratégie nationale de réduction de la violence à l'égard des femmes 2025–2029.

**Leçons tirées:**

Le cadre méthodologique RESPECT peut être adapté aux différents contextes locaux par le biais de la personnalisation et du renforcement des capacités, mais aussi en veillant à obtenir l'appui initial du gouvernement, en disposant d'un personnel spécialisé, en attribuant des ressources et en utilisant les relations établies avec les principales parties prenantes. L'amélioration de la recherche, du suivi et de l'évaluation est cruciale pour promouvoir des processus décisionnels étayés par des faits, tout comme l'implication des communautés et la collaboration multilatérale. L'ONU peut contribuer à ces efforts.

## Resumen

**Situación:**

La violencia contra las mujeres es un problema sanitario mundial. Para prevenirla de manera eficaz se necesitan estrategias adaptadas a las circunstancias locales.

**Enfoque:**

La Organización Mundial de la Salud en Indonesia, en colaboración con el Fondo de Población de las Naciones Unidas (ONU), ONU Mujeres, el Fondo de las Naciones Unidas para la Infancia, el Programa de las Naciones Unidas para el Desarrollo y el Programa Conjunto de las Naciones Unidas sobre el VIH/SIDA, emprendió iniciativas de capacitación para introducir RESPECT como marco basado en evidencias para abordar la violencia contra las mujeres. Las iniciativas incluyeron la participación de las partes interesadas, el desarrollo de módulos, formación de sensibilización, un taller y sesiones de evaluación. En estas sesiones se presentaron las estrategias de RESPECT a funcionarios del gobierno, oficiales de las Naciones Unidas y representantes de la comunidad, se trazaron las mejores prácticas y se desarrollaron planes de acción.

**Marco regional:**

Indonesia tiene una carga considerable de violencia contra las mujeres, a pesar de las numerosas leyes y políticas para contrarrestarla. La encuesta sobre violencia contra las mujeres realizada en Indonesia en 2021 mostró que el 26,1% (3341/12 800) de las mujeres de entre 15 y 64 años habían sufrido violencia a lo largo de su vida, y el 8,7% (1114/12 800) en el último año.

**Cambios importantes:**

Las iniciativas para introducir RESPECT llevaron a conseguir el apoyo del gobierno. Además, el Director para la Protección de los derechos de la mujer contra la violencia doméstica y la vulnerabilidad se comprometió verbalmente a integrar RESPECT en el Plan nacional de desarrollo 2025-2029 y en la Estrategia nacional para reducir la violencia contra la mujer.

**Lecciones aprendidas:**

Se puede adaptar RESPECT a los contextos locales mediante la personalización y el desarrollo de capacidades, y garantizando el apoyo inicial del gobierno, la dedicación de personal, la asignación de recursos y el uso de las relaciones establecidas con las principales partes interesadas. La mejora de la investigación, el seguimiento y la evaluación son vitales para promover la toma de decisiones basada en evidencias, junto con la participación de la comunidad y la colaboración de múltiples partes interesadas. La ONU puede facilitar estos esfuerzos.

## ملخص

**المشكلة:**

العنف ضد المرأة هو مشكلة صحية عالمية. ويتطلب منع هذا العنف بشكل فعال استراتيجيات مكيفة محليًا.

**لأسلوب:**

بالتعاون مع صندوق الأمم المتحدة للسكان، وهيئة الأمم المتحدة للمرأة، ومنظمة الأمم المتحدة للطفولة، وبرنامج الأمم المتحدة الإنمائي، وبرنامج الأمم المتحدة المشترك الخاص بفيروس نقص المناعة البشرية/الإيدز، أطلقت منظمة الصحة العالمية في إندونيسيا، مبادرات لبناء القدرات لإدخال RESPECT كإطار عمل قائم على الأدلة لمواجهة العنف ضد المرأة. وتضمنت المبادرات إشراك أصحاب المصلحة، وتطوير الوحدات، والتدريب على التوعية، وورشة عمل، وجلسات تقييم. قدمت هذه الجلسات استراتيجيات RESPECT للمسؤولين الحكوميين، وموظفي الأمم المتحدة وممثلي المجتمع، وحددت أفضل الممارسات ووضعت خطط العمل.

**المواقع المحلية:**

تحمل إندونيسيا إرثًا ثقيلاً من العنف ضد المرأة، على الرغم من وجود قوانين وسياسات عديدة لمكافحته. أظهر مسح العنف ضد المرأة لعام 2021 في إندونيسيا أن %26.1 (3341/12800) من النساء اللاتي تتراوح أعمارهن بين 15 و64 عامًا قد تعرضن للعنف في حياتهن، بينما تعرض %8.7 (1114/12800) منهن للعنف في العام الماضي.

**التغيّرات ذات الصلة:**

أدت مبادرات إطلاق RESPECT إلى تأمين الدعم الحكومي، كما قدم مدير مجلس "حماية حقوق المرأة من العنف المنزلي والضعف" التزامًا شفهيًا بدمج RESPECT في خطة التنمية الوطنية للفترة 2025 إلى 2029، والاستراتيجية الوطنية للحد من العنف ضد النساء.

**الدروس المستفادة:**

يمكن تكييف RESPECT مع الأوضاع المحلية من خلال التخصيص وبناء القدرات، وضمان الدعم الحكومي الأولي، وفرق العمل المكرّسة، وتخصيص الموارد، والاستفادة من العلاقات مع أصحاب المصلحة الرئيسيين. إن كل من البحث المُحسّن، والرصد، والتقييم تعد عوامل حيوية لتعزيز اتخاذ القرارات القائمة على الأدلة، إلى جانب مشاركة المجتمع، والتعاون بين العديدين من أصحاب المصلحة. ويمكن للأمم المتحدة تسهيل هذه الجهود.

## 摘要

**问题:**

暴力侵害女性行为是一个全球性健康问题。有效防止这种暴力行为需要因地制宜地制定战略。

**方法:**

世界卫生组织驻印度尼西亚办事处与联合国 (UN) 人口基金、联合国妇女署、联合国儿童基金会、联合国开发计划署和联合国艾滋病联合规划署合作，发起了能力建设倡议，将“尊重”作为解决暴力侵害女性问题的循证框架。这些举措包括邀请利益相关者参与、模块开发、性别敏感性训练以及召开研讨会和评估会议。通过这些会议向政府官员、联合国官员和社区代表介绍了“尊重”战略，详细讲解了最佳做法并制定了行动计划。

**当地状况:**

尽管印度尼西亚已制定了许多法律和政策来打击暴力侵害女性行为，但这个问题对该国政府来说仍是一个沉重的负担。2021 年印度尼西亚暴力侵害女性行为相关调查显示，15 至 64 岁女性中有 26.1% (3,341/12,800) 在其一生中曾遭到暴力侵害，而有 8.7% (1,114/12,800) 在过去一年中遭到过暴力侵害。

**相关变化:**

推崇“尊重”的举措获得了政府的支持，保护女性和弱势群体免受家暴行为侵害的权益保护局局长口头承诺会将“尊重”纳入 2025-2029 年国家发展计划和国家减少暴力侵害女性行为战略。

**经验教训:**

通过确保最初获得政府支持、部署专职人员、合理分配资源和利用与主要利益相关者之间的既定关系，在进行定制化和能力建设后，可根据当地情况制定“尊重”框架。改进研究、监测和评估方法对促进制定循证决策、提高社区参与度和加强多方利益相关者合作至关重要。联合国可致力于进一步改进这些方法。

## Резюме

**Проблема:**

Насилие в отношении женщин представляет собой глобальную проблему в области здравоохранения. Эффективное предотвращение такого насилия требует адаптированных к местным условиям стратегий.

**Подход:**

Всемирная организация здравоохранения в Индонезии в сотрудничестве с Фондом Организации Объединенных Наций (ООН) в области народонаселения, организацией «ООН-женщины», Детским фондом ООН, Программой развития ООН и Объединенной программой ООН по ВИЧ/СПИДу приступила к реализации инициатив по наращиванию потенциала с целью внедрения программы RESPECT в качестве научно обоснованной системы борьбы с насилием в отношении женщин. В число инициатив входили: привлечение заинтересованных сторон, разработка модулей, тренинги по повышению осведомленности, семинар и совещания по оценке ситуации. На этих совещаниях правительственные чиновники, сотрудники ООН и представители общин знакомились со стратегиями RESPECT, изучали передовые практические методы и разрабатывали планы действий.

**Местные условия:**

Несмотря на многочисленные законы и стратегии, направленные на борьбу с насилием в отношении женщин, в Индонезии наблюдается значительное количество случаев насилия в отношении женщин. По результатам проведенного в 2021 году в Индонезии исследования насилия в отношении женщин, 26,1% (3341/12 800) женщин в возрасте 15–64 лет подвергались насилию в течение своей жизни, а 8,7% (1114/12 800) – в течение последнего года.

**Осуществленные перемены:**

Инициативы по внедрению системы RESPECT способствовали получению поддержки со стороны правительства, и директор управления по защите прав женщин от домашнего насилия и уязвимости дал устное обещание включить систему RESPECT в Национальный план развития на 2025–2029 годы и Национальную стратегию по сокращению насилия в отношении женщин.

**Выводы:**

Система RESPECT может быть адаптирована к местным условиям путем адаптации и наращивания потенциала, а также путем обеспечения первоначальной государственной поддержки, выделения специального персонала, выделения ресурсов и использования сложившихся отношений с ключевыми заинтересованными сторонами. Для содействия принятию решений на основе фактических данных жизненно необходимы более эффективные исследования, мониторинг и оценка, а также вовлечение сообществ и сотрудничество с различными заинтересованными сторонами. Содействовать этим усилиям может ООН.

## Introduction

Violence against women is a global health problem rooted in gender inequality.^1^ Preventing violence against women in Indonesia is a distinct challenge: despite the existence of laws and policies aimed at eliminating violence against women, one in four women in the country still reports experiencing violence, based on the latest survey on violence against women in 2021.^2^ In 2019, the World Health Organization (WHO) and 13 other United Nations (UN) agencies and funders introduced RESPECT, an evidence-based framework that provides strategies for preventing violence against women.^3^ Although RESPECT has been piloted in several and low- and middle-income countries and has gained considerable support, the framework does not give details of how the strategies can be adapted to each country’s context.^4^ The original RESPECT documents also lack studies representing Indonesian women, as most evidence originates from high-income countries, with few studies from South-East Asia.^3^ Moreover, presenting RESPECT as an evidence-based framework is a challenge when advocating to decision-makers who may not have a research-oriented perspective, particularly in securing their commitment to evidence-informed decision-making.^5^^,^^6^

## Local setting

Indonesia, with a population of more than 270 million people, has a substantial burden of violence against women. The 2021 Indonesian survey on violence against women, which included a representative sample of 12 800 women, showed that 26.1% (3341) of women aged 15–64 years had experienced violence in their lifetime, with 8.7% (1114) experiencing violence in the past year.^2^ Data on violence against women in Indonesia are collected from three main sources: quinquennial surveys, an online information system and an annual report.^1^^,^^2^^,^^7^^,^^8^ However, bureaucratic challenges, varying standards, and limited resources have caused data inconsistencies and reliability issues.^8^

Regulations on violence against women in Indonesia include national laws, government and presidential decrees, ministerial regulations and regional rules. After ratifying the Convention on the Elimination of All Forms of Discrimination Against Women in 1984 and the Convention on the Rights of the Child in 1990, Indonesia enacted several laws related to violence against women, such as the Domestic Violence Law (23/2004), Child Protection Law (35/2014), Anti-Trafficking Law (21/2007), Law on Sexual Violence Crimes (12/2022) and the new criminal code (1/2023). The Ministry of Women Empowerment and Child Protection has the main mandate on violence against women and coordinates with other institutions.^7^^,^^9^

## Approach

To introduce RESPECT as an evidence-based framework to the Indonesian government, WHO Indonesia, in collaboration with UN Women, United Nations Population Fund (UNFPA), United Nations Children’s Fund (UNICEF), United Nations Development Programme (UNDP), and Joint United Nations Programme on HIV/AIDS (UNAIDS), initiated capacity-building initiatives including stakeholder engagement, module development, sensitization training, a workshop on RESPECT and an evaluation session (Box 1). These activities were led by UN officers and supported by consultants in collaboration with the Ministry of Women Empowerment and Child Protection. The approach was based on previous programmatic experiences in Indonesia and the implementation of RESPECT in other countries in the WHO South-East Asia Region, where capacity-building activities were used.^4^^,^^10^^,^^11^

Box 1 **Capacity-building process to introduce the RESPECT framework, Indonesia, 2023**

***Preparatory dialogue and securing government support (January–May 2023)***

- Held initial dialogues with the UN and key ministries, including Ministry of Women Empowerment and Child Protection and Ministry of National Development Planning;
- Aligned discussions with the government’s focus on the new law on sexual violence crimes;
- Used UN officers within Ministry of Women Empowerment and Child Protection for internal advocacy;
- Built on Ministry of Women Empowerment and Child Protection’s prior experience with a UNICEF-supported initiative; and
- Secured endorsement from Ministry of Women Empowerment and Child Protection as the primary government implementer.

***Module development (July 2023) and material preparation***

- Hired two consultants on violence against women in July 2023 to create the capacity-building module;
- Gathered relevant Indonesian laws, regulations, grey literature and reports (started in the first quarter of 2023);
- Completed the translation of selected RESPECT guidelines from English to Indonesian; and
- Designed the module as a living document to incorporate stakeholder input, aligned with the seven RESPECT strategies.

***Sensitization training (August 2023)***

- Introduced the seven RESPECT strategies;
- Collected feedback for the module; and
- Prepared participants to serve as gender champions to actively encourage discussions during the main workshop.

***Main workshop (September 2023)***

- Introduced the seven RESPECT strategies;
- Mapped best practices for the prevention of violence against women;
- Developed theories of change; and
- Formulated realistic follow-up plans for integrating RESPECT into policies and programmes.

***Evaluation session (November 2023)***

- Secured an oral commitment to integrate RESPECT as an evidence-based guiding principle into the National Medium Term Development Plan for 2025–2029 and the National Strategy for Reducing Violence Against Women. Further implementation follow-up is needed; and
- Symbolized support for adopting global guidance within Ministry of Women Empowerment and Child Protection’s technical capacity.

UN: United Nations; UNICEF: United Nations Children’s Fund.

RESPECT was not altered as a guideline; however, through a collaborative stakeholder approach a capacity-building module for a 3-day training course was developed. The first day focused on violence against women and intersectionality; the second day covered the human rights framework and an introduction to RESPECT; and the third day addressed theory of change and action plans. The materials included all relevant elements from RESPECT’s 10-step implementation framework, such as understanding violence against women; assessing risks and enablers; gathering evidence for the seven RESPECT strategies; developing a theory of change in relation to the seven strategies; strengthening enabling environments; and emphasizing monitoring, evaluation and scaling up of effective practices.^3^

To secure government trust, initial dialogues (January–May 2023) were held between UN officers and key ministries, including the Ministry of Women Empowerment and Child Protection and Ministry of National Development Planning. The timing aligned with the government’s focus on the new law on sexual violence crimes.^12^ Dedicated UN officers within the Ministry of Women Empowerment and Child Protection, particularly from UNFPA, facilitated internal advocacy. The ministry’s previous experience with a UNICEF-supported capacity-building initiative on violence against children also increased its openness to global guidance.^11^

In July 2023, WHO Indonesia recruited two consultants on violence against women to create the capacity-building module and conduct the training. Earlier, UN and government officers gathered relevant Indonesian laws, regulations, grey literature and reports from the ministry, National Commission on Violence against Women, UN agencies and other organizations to support our advocacy dialogue and ensure that the RESPECT framework aligned with local strategies. This preparation also helped us tailor the capacity-building module to fit the specific needs and context of Indonesia.

The RESPECT implementation guidelines were translated into Indonesian. The capacity-building module was designed as a living document and allowed stakeholders to provide input based on their expertise. The module was aligned with the seven RESPECT strategies (relationship skills strengthened; empowerment of women; services ensured; poverty reduced; environments made safe; child and adolescent abuse prevented; and transformed attitudes, beliefs and norms). For instance, success stories from the Better Sexual and Reproductive Health and Rights for All in Indonesia (BERANI) I project (2018–2023) were incorporated into the document to demonstrate the application of RESPECT strategies to enhance sexual and reproductive health and rights for women and young people. Specifically, training and essential services were provided for 2371 survivors of violence against women in integrated service centres; comprehensive sexuality education and sexual and reproductive health and rights outreach programmes raised awareness among 29 918 adolescents; and collaboration with government institutions and advocacy efforts influenced the creation of 21 policies and advocacy strategies promoting sexual and reproductive health and rights.^13^

Using the capacity-building module, two 3-day sessions were conducted in Jakarta (Box 1). The sensitization training was aimed to introduce the seven RESPECT strategies, collect feedback for the module and prepare participants to serve as gender champions who could actively encourage discussions during the main workshop. The main workshop focused on mapping best practices on the prevention of violence against women in alignment with the seven RESPECT strategies, developing a theory of change, and formulating realistic follow-up plans for the government and relevant organizations to integrate RESPECT principles into policies and programmes.

The sensitization training (23–25 August 2023) included 30 representatives from the government, UN agencies and community organizations. A coordination meeting on 4 September 2023 with key stakeholders at the Ministry of Women Empowerment and Child Protection set the stage for the workshop. The main workshop (13–15 September 2023) comprised 67 participants from 37 organizations, including gender champions, government policy-makers, UN officers and representatives of nongovernmental organizations.^10^ Ministry of Women Empowerment and Child Protection in collaboration with UN agencies invited stakeholders representing the seven areas of the RESPECT prevention strategies, including participants working in health care, policy, gender, economics, social affairs, law enforcement, education, community organizations, religion, academia, business, transportation, philanthropy and development. Key grassroots organizations representing vulnerable women, young women, disability groups, women living with human immunodeficiency virus, transgender women and faith-based organizations enriched discussions with intersectional perspectives.

## Relevant changes

Despite limited time and the absence of studies on impact validity, the introduction of RESPECT through capacity-building initiatives secured initial government support. Potential exists to integrate RESPECT’s seven strategies into the policy and programmatic initiatives of the Ministry of Women Empowerment and Child Protection. The evaluation session on 27 November 2023 led to a verbal commitment to use RESPECT as an evidence-based guiding principle in the upcoming National Medium-Term Development Plan for 2025–2029 and the National Strategy for Reducing Violence Against Women.^10^ Although the plan has not yet been executed and further implementation follow-up is needed, this commitment symbolizes an approval for adopting global guidance within the technical capacity of the Ministry of Women Empowerment and Child Protection.

## Lessons learnt

Adapting WHO and UN guidelines to local contexts requires systematic approaches that blend customization with capacity-building initiatives.^5^ Initiating the first step of introducing RESPECT as an evidence-based framework required a comprehensive advocacy process to generate support. Using established relationships with key stakeholders is vital, particularly for complex topics such as violence against women that require multistakeholder collaboration. Clear prioritization, dedicated personnel and continuous resource allocation are necessary to support RESPECT adaptation efforts (Box 2).

Box 2 **Summary of main lessons learnt**

- Adapting the RESPECT framework to the local contexts is feasible through systematic approaches that combine customization and capacity-building initiatives, supported by clear prioritization, dedicated personnel, resource allocation and established relationships with key stakeholders.
- Introducing the RESPECT framework requires galvanizing government support through a comprehensive advocacy process that includes stakeholder engagement and capacity-building sessions.
- Enhancing research, and monitoring and evaluation combined with meaningful community engagement and multistakeholder collaboration are essential to promote evidence-informed decision-making in the prevention of violence against women.

Progress on the RESPECT initiative was coordinated by a dedicated officer within WHO and supported by other UN agency officers and experienced consultants with previous experience in the National Commission on Violence against Women, who provided valuable insider perspectives. Although RESPECT Indonesia experienced limited progress from its launch in 2019, dedicated personnel revitalized the initiative in 2023, underscoring the importance of prioritization.^10^ A clear description of contributions from each organization is essential to drive interagency collaboration.

WHO Indonesia capitalized on the established relationships of other UN agencies, particularly UNFPA, UN Women and UNICEF, with the Ministry of Women Empowerment and Child Protection, and secured full support from the Director for the Protection of Women’s Rights against Domestic Violence and Vulnerability. Multiple UN agencies contributed to the project’s costs: WHO covered consultant and venue costs; UN Women covered merchandise, translators and sign language interpreters; UNAIDS funded cash disbursements; and UNFPA financed the evaluation workshop. The total amount was 41 750 United States dollars.

While RESPECT provides evidence from randomized controlled trials on effective prevention of violence against women, a realistic adaptation of the capacity-building module sometimes relies on case studies and success stories from government and UN programmes sourced from unpublished reports and internal evaluations.^3^^,^^14^ Most programmes on violence against women in Indonesia are not designed as research endeavours, which highlights the need to strengthen research in monitoring and evaluation processes to enhance evidence-informed decision-making.

Although different stakeholders and community members participated in the RESPECT sensitization training and workshop, their involvement must extend beyond these events. The UN plays a crucial role in fostering community engagement, ensuring participatory and democratic advocacy for prevention of violence against women, and coordinating efforts among key stakeholders. RESPECT functioned as a central coordinating mechanism, bringing together multiple stakeholders towards the common goal of preventing violence against women, with the UN facilitating meaningful engagement and discussion.^15^
